# Supplementary material for: A prospective cohort analysis from Germany shows transition into adulthood is an underestimated vulnerable period for children with overweight/obesity
Source: Commun Med (Lond). 2025 Oct 30;5:447. doi: 10.1038/s43856-025-01197-8 (PMC12575610; doi:10.1038/s43856-025-01197-8)
Supplement: Supplementary file 2 — Supplementary Material [file 43856_2025_1197_MOESM2_ESM.pdf]

Supplementary Material

A prospective cohort analysis from Germany shows transition into adulthood is an underestimated vulnerable period for children with overweight/obesity

Johannes Riedel[1], Natascha Genge[1], Klara Meyer, MD[1], Eric Wenzel, MD[1,2,3], Elena Sergeyev, MD[1], Katja Mühlberg, MD[2,4], Sabine Steiner, MD[2,5], Wieland Kiess, MD[1,6], Roland Pfäffle, MD[1,3], Matthias Blüher, MD[2,7], Antje Körner, MD[1,2,3,6 #], Robert Stein, MD[1,2,3 #]

[1] University of Leipzig, Medical Faculty, University Hospital for Children & Adolescents, Center for Pediatric Research Leipzig, Germany
[2] Helmholtz Institute for Metabolic, Obesity and Vascular Research (HI-MAG) of the Helmholtz Zentrum München at the University of Leipzig and University Hospital Leipzig, Germany
[3] German Center for Child and Adolescent Health (DZKJ), partner site Leipzig/Dresden, Leipzig, Germany
[4] University Hospital Leipzig, Department of Internal Medicine, Neurology and Dermatology, Division of Angiology, Leipzig, Germany
[5] Medical University Vienna, Department of Medicine II, Division of Angiology, Vienna, Austria
[6] LIFE–Leipzig Research Center for Civilization Diseases, University of Leipzig, Germany
[7] University of Leipzig, Medical Faculty, Department of Endocrinology, Nephrology und Rheumatic diseases, Leipzig, Germany

# Authors contributed equally

Address correspondence to:
Prof. Antje Körner, MD
Helmholtz Institute for Metabolic, Obesity and Vascular Research (HI-MAG)
of the Helmholtz Zentrum München
at the University of Leipzig and University Hospital Leipzig, Germany
Email: Antje.Koerner@medizin.uni-leipzig.de
Phone: +49-341-9726500

Table of contents

Figure S1: Health burden of young adults with childhood onset obesity……………..……………………………3
Figure S2: Perceived health burden of young adults with childhood onset obesity……………………………….4
Figure S3: Timing of transition…………………………………………………………………………………….5
Figure S4: Treatment continuation after transition in young adulthood…………………………………………...6
Figure S5: Compliance of young adults with childhood onset obesity…………………………………………….7

Table S1: Characteristics of successfully recontacted participants at their last childhood visit…………………...8
Table S2: Treatment continuation after transition and BMI dynamics…………………………………………….9
Table S3: Past weight loss attempts…………………………………………………………………………….10

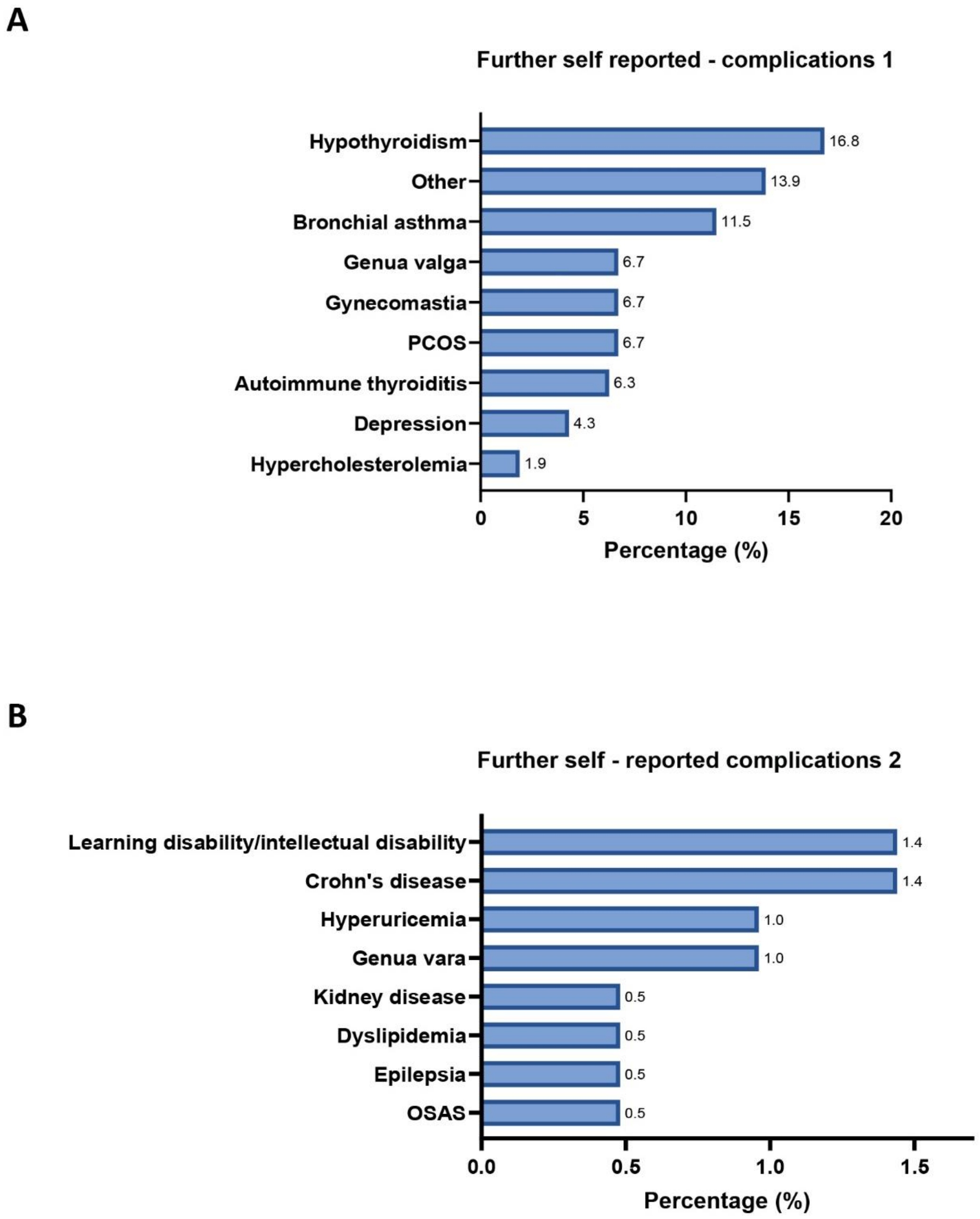

Figure S1: Health burden of young adults with childhood onset obesity. Depicted are the responses to a questionnaire as indicated in the graph headings (N = 209).

Abbreviations: *N*, number of subjects; *OSAS*, obstructive sleep apnea syndrome; *PCOS*, polycystic ovary syndrome.

A

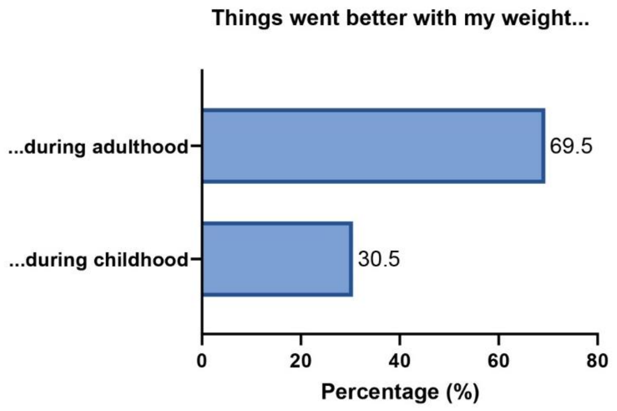

B

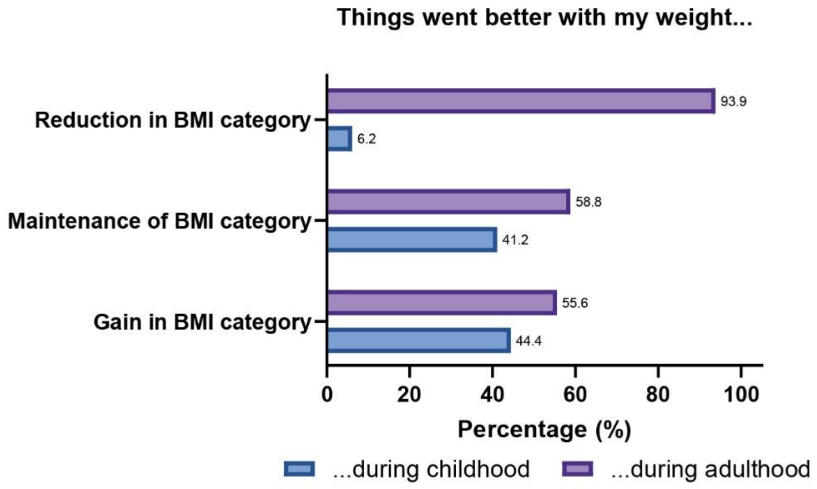

Figure S2: Perceived health burden of young adults with childhood onset obesity. Most adults with childhood onset obesity feel that "things went better with their weight" during adulthood (A), even if they had an unfavorable change in BMI class ("Gain in BMI category", B); N = 203 subjects.

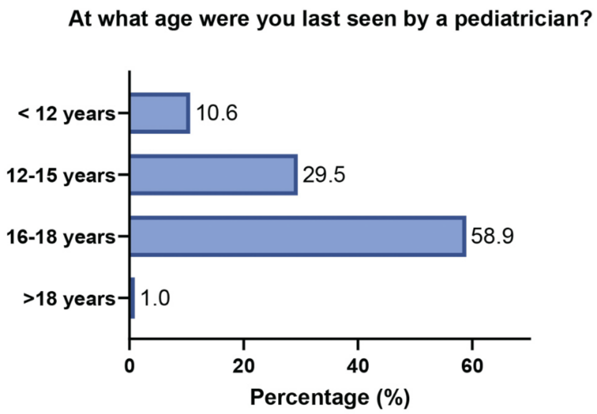

Figure S3: Timing of transition. Depicted are the responses to the indicated question in the graph heading (N = 207 subjects).

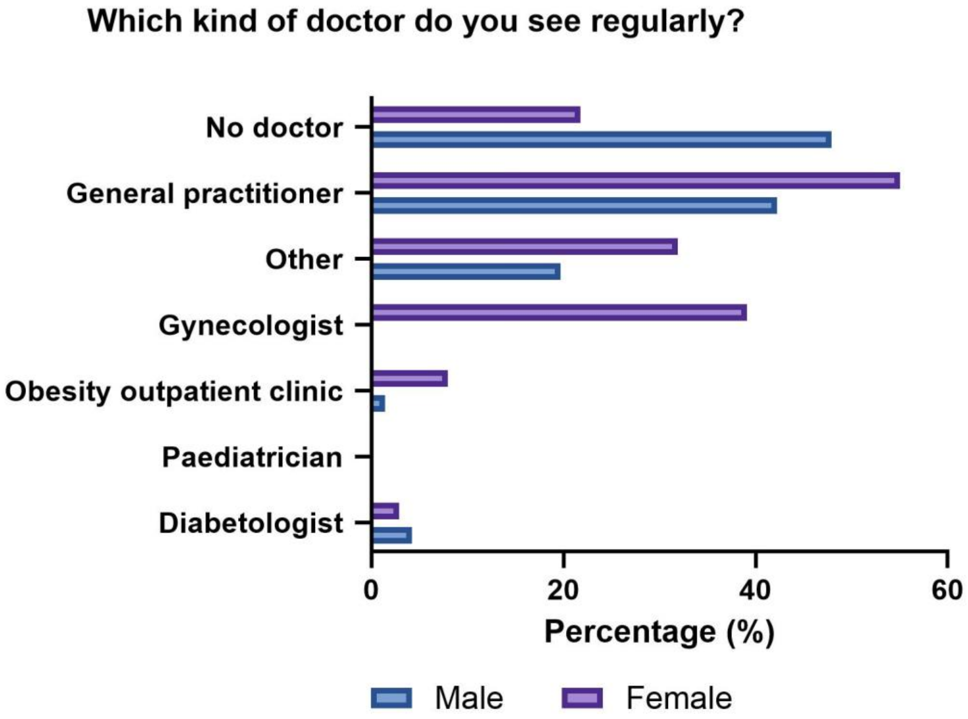

Figure S4: Treatment continuation after transition in young adulthood. Women were more likely to see a doctor on a regular basis (N = 209 subjects).

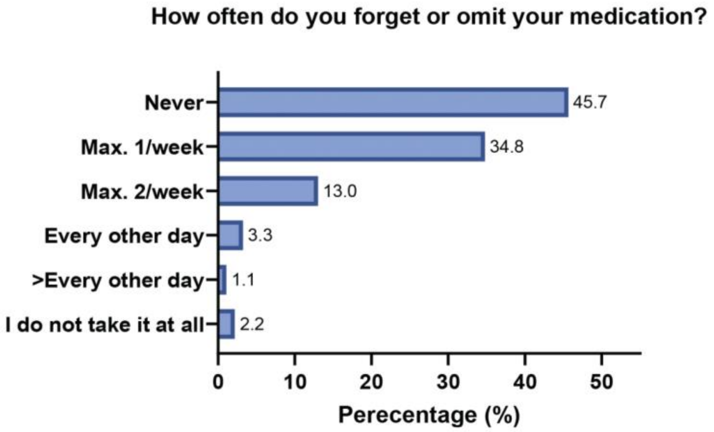

Figure S5: Compliance of young adults with childhood onset obesity. Only participants who reported regular intake of medication were asked to answer the indicated question (N = 92 subjects).

Table S1: Characteristics of successfully recontacted participants at their last childhood visit.
Differences between groups were tested by two-tailed student's t-test for continuous variables and X^2-test for categorial variables. Abbreviations: *N*, number of subjects.

		**Participated in follow-up visit (N=209)**	**Not willing to participate (N=148)**	**p-value**
Age in years	Mean (range)	13.24 (2-17)	13.36 (4 -17)	0.27
Sex	N male (percentage)	71 (33.97%)	71 (47.97%)	0.008
	N female (percentage)	138 (66.03%)	77 (52.03%)	
BMI SDS	Mean (range)	2.59 (0.91-5.03)	2.48 (0.36-4.71)	0.13

Table S2: Treatment continuation after transition and BMI dynamics. Participants were asked at adulthood follow-up: “Which kind of doctor do you see regularly?” Those with regular doctor visits were seen by their GPs, gynecologists, obesity outpatient clinic, diabetologists, and/or others. *N*, number.

	**Reduction in BMI category**	**Maintenance of BMI category**	**Gain in BMI category**
**Regular doctor visits during adulthood**	**N answers (%)**	**N answers (%)**	**N answers (%)**
Yes, N=145 (69.38 %)	39 (60.0 %)	82 (75.93 %)	24 (66.67 %)
No, N=64 (30.62 %)	26 (40.0 %)	26 (24.07 %)	12 (33.33 %)

Table S3: Past weight loss attempts
Participants were asked at adulthood follow-up: "Have you ever tried to lose weight and if so, how?" *N*, number.

**Weight loss attempt**	**All participants (N=209)**	**Reduction in BMI category (N=65)**	**Maintenance of BMI category (N=108)**	**Gain in BMI category (N=36)**
	**N answers (%)**	**N answers (%)**	**N answers (%)**	**N answers (%)**
Weight-loss medication/appetite suppressants*	10 (4.78%)	3 (30%)	3 (30%)	4 (40%)
GLP-1 receptor agonists	5 (2.39%)	1 (20%)	3 (60%)	1 (20%)
Participation in a structured lifestyle intervention program**	55 (26.32%)	18 (32.73%)	29 (52.73%)	8 (14.55%)
Dietary change with the support of a nutritionist/dietitian	54 (25.84%)	12 (22.22%)	34 (62.96%)	8 (14.81%)
Dietary change on your own initiative	138 (66.03%)	51 (36.96%)	65 (47.10%)	22 (15.94%)
Regular physical activity with a fitness coach/professional support	25 (11.96%)	7 (28.0%)	13 (52.0%)	5 (20.0%)
Regular physical activity on your own initiative	137 (65.55%)	47 (34.31%)	66 (48.18%)	24 (17.52%)
Weight-loss app	55 (26.32%)	15 (27.27%)	32 (58.18%)	8 (14.54%)
Several-week inpatient rehabilitation program	33 (15.79%)	6 (18.18%)	25 (75.76%)	2 (6.06%)
Bariatric surgery (sleeve gastrectomy)	6 (2.87%)	0 (0%)	6 (100%)	0 (0%)
Bariatric surgery (gastric bypass)	2 (0.96%)	2 (100%)	0 (0%)	0 (0%)
Other***	6 (2.87%)	0 (0%)	5 (83.33%)	1 (16.67%)
No	16 (7.66%)	3 (18.75%)	8 (50.0%)	5 (31.25%)

* Refers to weight-loss medication other than GLP-1 receptor agonists or not further specified; ** Information about lifestyle intervention programs were retrieved from the questionnaire and medical records; *** Other: Work-related physical activity (N=3); Covid-19 pandemic (N=1); change of environment (N=1); bulimia (N=1)
