# Supplementary material for: A prospective cohort analysis from Germany shows transition into adulthood is an underestimated vulnerable period for children with overweight/obesity
Source: Commun Med (Lond). 2025 Oct 30;5:447. doi: 10.1038/s43856-025-01197-8 (PMC12575610; doi:10.1038/s43856-025-01197-8)
Supplement: Supplementary file 3 — Description of Additional Supplementary Files [file 43856_2025_1197_MOESM3_ESM.pdf]

Description of Additional Supplementary Files

File name: Supplementary Data 1

Description: Questionnaires developed by the research team

File name: Supplementary Data 2

Description: Summary source data for main figures 2-5
